# *P*. *falciparum* and *P*. *vivax* Epitope-Focused VLPs Elicit Sterile Immunity to Blood Stage Infections

**DOI:** 10.1371/journal.pone.0124856

**Published:** 2015-05-01

**Authors:** David C. Whitacre, Diego A. Espinosa, Cory J. Peters, Joyce E. Jones, Amy E. Tucker, Darrell L. Peterson, Fidel P. Zavala, David R. Milich

**Affiliations:** 1 Vaccine Research Institute of San Diego, San Diego, California, United States of America; 2 VLP Biotech, Inc., San Diego, California, United States of America; 3 Department of Molecular Microbiology and Immunology, Malaria Research Institute, Bloomberg School of Public Health, Johns Hopkins University, Baltimore, Maryland, United States of America; 4 Department of Biochemistry, Virginia Commonwealth University, Richmond, Virginia, United States of America; Queensland Institute of Medical Research, AUSTRALIA

## Abstract

In order to design *P*. *falciparum* preerythrocytic vaccine candidates, a library of circumsporozoite (CS) T and B cell epitopes displayed on the woodchuck hepatitis virus core antigen (WHcAg) VLP platform was produced. To test the protective efficacy of the WHcAg-CS VLPs, hybrid CS *P*. *berghei/P*. *falciparum* (Pb/Pf) sporozoites were used to challenge immunized mice. VLPs carrying 1 or 2 different CS repeat B cell epitopes and 3 VLPs carrying different CS non-repeat B cell epitopes elicited high levels of anti-insert antibodies (Abs). Whereas, VLPs carrying CS repeat B cell epitopes conferred 98% protection of the liver against a 10,000 Pb/Pf sporozoite challenge, VLPs carrying the CS non-repeat B cell eptiopes were minimally-to-non-protective. One-to-three CS-specific CD4/CD8 T cell sites were also fused to VLPs, which primed CS-specific as well as WHcAg-specific T cells. However, a VLP carrying only the 3 T cell domains failed to protect against a sporozoite challenge, indicating a requirement for anti-CS repeat Abs. A VLP carrying 2 CS repeat B cell epitopes and 3 CS T cell sites in alum adjuvant elicited high titer anti-CS Abs (endpoint dilution titer >1x10^6^) and provided 80–100% protection against blood stage malaria. Using a similar strategy, VLPs were constructed carrying *P*. *vivax* CS repeat B cell epitopes (WHc-Pv-78), which elicited high levels of anti-CS Abs and conferred 99% protection of the liver against a 10,000 Pb/Pv sporozoite challenge and elicited sterile immunity to blood stage infection. These results indicate that immunization with epitope-focused VLPs carrying selected B and T cell epitopes from the *P*. *falciparum* and *P*. *vivax* CS proteins can elicit sterile immunity against blood stage malaria. Hybrid WHcAg-CS VLPs could provide the basis for a bivalent *P*. *falciparum/P*. *vivax* malaria vaccine.

## Introduction

Malaria is an important tropical parasitic disease that kills more people than any other communicable disease with the exception of tuberculosis. The causative agents in humans are four species of Plasmodium protozoa: *P*. *falciparum*, *P*. *vivax*, *P*. *ovale* and *P*. *malariae*. Of these, *P*. *falciparum* (Pf) is the most lethal. The vast majority of deaths occur among young children in Africa. *P*. *vivax* is the most prevalent species outside of sub-Saharan Africa and responsible for approximately 50% of all malaria cases worldwide [1]. Malaria is a public health problem today in more than 106 countries, inhabited by a total of 3.4 billion people-50% of the world’s population. Worldwide prevalence of the disease is estimated to be on the order of 135–287 million clinical cases each year. Mortality due to malaria is estimated to be in the range of 473,000–789,000 each year [2]. The *P*. *falciparum* malaria parasite has 14 chromosomes, an estimated 5,300 genes (many of which vary extensively between strains) and a complex four-stage life cycle as it passes from a mosquito vector to humans and back again. Furthermore, the natural *P*. *falciparum* infection does not result in immunity, and partial immunity occurs only after years of recurring infections and illnesses. Therefore, a vaccine must out perform the immune response to the natural infection. This complexity and the lack of suitable animal models has impeded vaccine development against both *P*. *falciparum* and *P*. *vivax*.

All stages of the *P*. *falciparum* malaria life cycle have been targeted for vaccine development, however, only preerythrocytic stage (i.e., the circumsporozoite (CS) protein [3] and the multiepitope (ME)-thrombospondin-related adhesion protein (TRAP) [4]) immunogens have been shown to elicit significant clinical efficacy. Only one CS vaccine candidate has reached phase III clinical trials, known as RTS,S, which targets the CS protein's NANP repeat B cell epitopes and C-terminal T cell domains by fusing them to the hepatitis B surface antigen (HBsAg) [3]. RTS,S has been in development for over two decades and tested in multiple experimental and field trials [3,5–10]. In brief, protective efficacy for 3 doses of RTS,S formulated in a combination of three relatively potent adjuvants is reported as between 30 and 50% as judged by preventing clinical and severe malaria and the level of protection is dependent on malaria transmission intensity, age and time since vaccination [11]. Development of RTS,S is a significant achievement and demonstrates that a recombinant subunit vaccine containing only isolated B and T cell epitopes from a single CS protein delivered on a heterologous carrier can elicit protection in humans. However, it is generally acknowledged that “second generation” vaccines will be necessary for full implementation of a malaria vaccine intended for all at-risk populations (i.e., endemic populations, travelers to endemic regions and the military) [3]. The search for second generation preerythrocytic vaccine candidates has included use of the entire CS protein, addition of other preerythrocytic antigens, new adjuvants, DNA delivery, viral vectors, prime-boost strategies, etc., with little success to date [12]. The development of the RTS,S vaccine has concentrated on formulation optimization using the same antigen construct for over two decades.

Our CS epitope-focused approach has been to test multiple constructs on alternative carrier platforms, chosen because they are more immunogenic than the HBsAg, namely the hepadnavirus nucleocapsid proteins (i.e., HBcAg and WHcAg), to carry CS-derived B and T cell epitopes [13–15]. In the current study 2 CS repeat B cell epitopes (NANP-based and NVDP-based), singly or combined, were genetically inserted onto the WHcAg carrier as were 3 CS-derived non-repeat B cell epitopes and 1 to 3 well-defined human T cell domains. Hybrid VLPs were screened based on VLP self-assembly, expression level, antigenicity, immunogenicity and most importantly protective efficacy in an infectious *in vivo* model. Rodent malaria (*P*. *berghei*) parasites bearing the extended repeat region of the *P*. *falciparum* CS protein have been developed as an important preclinical tool for evaluating the efficacy of human CS protein-based vaccine candidates *in vivo* [16]. Chimeric Pb/Pf sporozoites are fully infectious in mice and can be neutralized by Abs and/or T cells specific for the portions of the *P*. *falciparum* CS protein replacing the *P*. *berghei* CS protein. Employing the WHcAg combinatorial VLP technology combined with the Pb/Pf hybrid sporozoite challenge system has allowed us to develop and test a variety of WHcAg-CS hybrid VLPs. Selected VLPs carrying Pf-specific CS repeat, but not non-repeat B cell epitopes were capable of eliciting sterile immunity against blood stage infection. Using a similar strategy we also developed a hybrid VLP carrying *P*. *vivax* CS repeat B cell epitopes and immunized mice challenged with Pb/Pv hybrid sporozoites [17] demonstrated full protection from blood stage malaria.

## Materials and Methods

### Animals

The (B10xB10.s) F1 mice used in VLP screening and immunogenicity evaluation were obtained from the breeding colony of the Vaccine Research Institute of San Diego (VRISD). The B6 mice used for protection studies were obtained from NCI (Fredrick, MD). The rabbits used for immunogenicity testing were New Zealand White rabbits obtained from ProSci Inc. (Poway, California). All animal care was performed according to National Institutes of Health standards as set forth in the *Guide for the Care and Use of Laboratory Animals* (2011). Animals at all facilities were monitored at least weekly.

### Ethics Statement

Experimental procedures involving F1 mice were carried out at Explora BioLabs (San Diego, CA), where they were housed, and were conducted by VRISD and VLP Biotech researchers under approval of the Explora BioLabs Institutional Use Committee (Protocol Number EB13-028, approved for the currently described studies). Experimental procedures involving B6 mice were carried out at Johns Hopkins University and were approved by the Institutional Animal Care and Use Committee of the Johns Hopkins University (Protocol Number MO13H123, approved for the currently described studies). The experiment involving rabbits was approved by ProSci Institutional Animal Care and Use Committee (paper protocol on file approved 11/5/2009). Humane endpoints were used: in the blood-stage challenge studies, mice were monitored daily and euthanized when they were infected by malaria parasites. Animals were euthanized by CO_2_ inhalation in accordance with the AMVA Guidelines on Euthanasia.

### Recombinant WHcAg hybrid VLP Construction

The WHcAg and hybrid WHcAg VLPs were expressed from the pUC-WHcAg vector expressing the full-length WHcAg protein codon optimized for expression in *E*. *coli* [18]. The sequence for WHcAg (accession NC_004177) was cloned into the pUC19 vector. For inserting heterologous B cell epitopes, *Eco*RI-*Xho*I restriction sites were engineered into the open reading frame between amino acids 78 and 79 of the core protein gene. The engineered restriction sites add a Gly-Ile-Leu linker on the N-terminal side and a Leu linker on the C-terminal side of the inserted epitopes. For fusing heterologous T cell epitopes, an *Eco*RV restriction site was engineered at the 3' end of the WHcAg gene, which adds an Asp-Ile linker between the WHcAg gene and the fused epitopes. Epitopes were cloned into the VLP gene using synthetic oligonucleotides comprising the desired epitope coding sequence and the appropriate engineered restriction sites. The WHc(C61S) point mutation to reduce carrier antigenicity was constructed by PCR using primer mismatches to create point mutations. All WHcAg constructs were transformed into Alpha-Select competent *E*. *coli* (Bioline USA, Inc.) and confirmed by DNA sequencing. Inserted B cell epitope sequences were exactly matched to the CSP sequence from clone 7G8 of *P*. *falciparum* used in the construction of the CS(Pf) or the VK210 repeat from Salvador I strain of *P*. *vivax* used in the construction of the Pb/Pv hybrid sporozoites, respectively. The inserted T cell epitopes had only one conservative mismatch, i.e., Cys 283 of the CS protein in the new Pb-Pf-CSP-CT hybrid sporozoites (see below and S1B Fig) is a Ser in the VLPs with the TH and 3T T cell epitope sequences (see S2A Fig).

### Purified Proteins and Synthetic Peptides

The VLP particles were expressed in Alpha-Select *E*. *coli* cells grown in Terrific Broth (Teknova, Hollister, CA). Cells were lysed by passage through an EmulsiFlex-C3 (Avestin, Ottawa, ON, Canada) and the lysate heated to 65°C for approximately 10 min, then clarified by centrifugation. The WHcAg particles were selectively precipitated by the addition of solid ammonium sulfate to approximately 45% saturation (277 g/L) and the precipitates were collected by centrifugation. Precipitated VLPs were redissolved in minimum buffer (10mM Tris, pH 8), dialized against the same buffer and applied to a Sepharose CL4B column (5x100cm). Finally, VLPs were formulated in 20mM Tris, pH8, 100mM NaCl. Endotoxin was removed from the core preparations by phase separation with Triton X-114 [19,20]. Briefly, the VLP solution was made 1% Triton X-114 and incubated at 4°C for 30 min with mixing, incubated at 37°C for 10 min, centrifuged at 20KxG for 10 min and the protein recovered in the upper phase. This was repeated for 4 extractions. The purified VLPs were 0.2um sterile-filtered, characterized and aliquoted. Characterization typically includes custom ELISA, native agarose gel electrophoresis, PAGE, heat stability, circular dichroism and dynamic light scattering as previously described [18,21].

Recombinant CS protein was produced from the CS27 IV C clone (MRA-272, MR4, ATCC Manassas, VA) obtained through the Malaria Research and Reference Reagent Resource Center (www.mr4.org) and deposited by Photini Sinnis [22]. The open reading frame was moved to the pQE-60 vector (Qiagen) and transformed into M15 *E*. *coli* cells (Qiagen). Integrity of the gene was confirmed by DNA sequencing before purification by standard methods. Briefly, LB medium, supplemented with 2 g/L glucose, 25 μg/ml Kanamycin and 50 μg/ml Ampicilin, was inoculated with a 1:40 dilution of overnight culture. Bacteria were grown at 37°C to an A600 of 0.8–1.0, isopropyl β-D-1-thiogalactopyranoside added to a concentration of 100 mg/L, grown 3 hours longer and harvested by centrifugation. Cells were suspended in lysis buffer (25 mM Tris, pH 8, 0.3 M NaCl, 10 mM imidazole) and lysed by a single passage through an Avestin EmulsiFlex-C3 (Ottawa,ON, Canada). The lysate was clarified by centrifugation at 48,000 X G for 30 min, and applied to a nickel column (BioRad Profinity IMAC). Unbound proteins were removed by elution with the lysis buffer, then bound proteins were eluted in the same buffer containing 100 mM imidazole. This procedure yielded approximately 10mg of pure protein per liter of cultured bacteria.

Synthetic peptides derived from the WHcAg or CS sequences were synthesized by Eton Biosciences (San Diego, CA).

### Immunizations and serology

Groups of mice were immunized intraperitoneally (i.p.) with the WHcAg hybrid VLPs (usually 10–20 μg) emulsified in incomplete Freund’s adjuvant (IFAd) for both antibody production and T cell experiments. The dose was varied when other adjuvants were used, i.e., saline (200 μg) and alum (100 μg). For antibody experiments, mice were bled retro-orbitally and sera pooled from each group. Periodically individual mouse sera were tested to confirm the fidelity of the pooled sera results. Anti-WHc and anti-insert immunoglobulin G (IgG) antibodies were measured in murine sera by an indirect solid-phase ELISA by using the homologous WHcAg (50 ng/well) or synthetic peptides (0.5 μg/well), representing the inserted sequence, as solid-phase ligands as described previously [15]. Serial dilutions of both test sera and preimmunization sera were made and the data are expressed as antibody titers representing the reciprocals of the highest dilutions of sera required to yield an optical density at 492 nm (OD 492) three times an equal dilution of preimmunization sera. IgG isotype-specific ELISAs were performed by using IgG1-, IgG2a-, IgG2b- and IgG3-specific peroxidase-labeled secondary antibodies (Southern Biotechnology, Birmingham, AL). Rabbits were immunized with WHcAg hybrid VLPs (200 μg in IFAd) and boosted either with 200 μg in saline or 100 μg in IFAd.

### IFA

Indirect immunofluorescence assays (IFA) using both live and air-dried sporozoites were used to characterize and titrate antibody responses. Briefly, for live-sporozoite IFAs, 40,000 parasites were incubated on ice with different sera dilutions. Sporozoites were then washed 3 times with cold PBS with 1% BSA, suspended in 0.2 ml and placed into the well of a Lab-Tek chambered coverglass (Thermo Scientific Nunc, Rochester, NY). The chamber was then spun at 300 x G for 2 min and, after discarding the supernatant, 0.2 ml of PBS with 4% Paraformaldehyde (Sigma, Saint Louis, MO) were added. Samples were incubated for 1 h at room temperature, washed 3 times with PBS and incubated with secondary antibody [AlexaFluor 488 F(ab’)2 fragment of goat anti-mouse IgG(H+L); 2 mg/ml; Invitrogen] for 30 min. Samples were then washed and green-fluorescent sporozoites were visualized using a Nikon Eclipse 90i fluorescent microscope. IFAs using air-dried sporozoites were performed as previously described [17].

### *In vitro* T cell cytokine assays

Spleen cells from groups of 3 each of (B10xB10.s) F1 mice were harvested and pooled 4–6 weeks after immunization with the various WHcAg hybrid VLPs. Spleen cells (5×10^5^) were cultured with varying concentrations of WHcAg, CS or synthetic peptides derived from WHcAg or CS protein. For cytokine assays, culture supernatants were harvested at 48 h for IL-2 determination and at 96 h for interferon-gamma (IFNγ) determination by ELISA. IFNγ production was measured by a two-site ELISA using mAb 170 and a polyclonal goat anti-mouse IFNγ (Genzyme Corp., Boston, MA).

### Development of *P*. *berghei* chimeric parasites expressing the C-terminal region of the *P*. *falciparum* CS protein (Pb/Pf-CSP-CT)

The new transgenic strain derived from *P*. *berghei* ANKA strain expressing the C-terminus of *P*. *falciparum* was generated using the plasmid pR-CSPfCT, which carries the C-terminal region of the *P*. *falciparum* CSP. This plasmid was derived from plasmid pIC-CSPfCT, which resulted from the replacement of the *P*. *berghei* CSP C-terminus with the C-terminal region of the 3D7 strain of *P*. *falciparum* CSP. Briefly, a 306-bp restriction fragment encompassing base pairs 715 to 1020 of the *P*. *berghei* CSP gene was excised from a modified version of pIC-CSwt [23] using the restriction enzymes *SexA*I and *Pac*I and then replaced with a fragment comprising the *P*. *falciparum* CSP C-terminal region (S1A Fig). The *P*. *falciparum* CSP C-terminus was excised as a 312-bp *SexA*I-*Pac*I restriction fragment from plasmid pPfCT (Genescript, Piscataway Township, NJ), synthesized to comprise the *P*. *falciparum* CSP C-terminal region. Thus, the CSP gene in the resulting plasmid, pIC-CSPPfCT, consists of the *P*. *berghei* N-terminal and repeat regions (base pairs 1 to 786) and the remainder of the *P*. *falciparum* CSP (base pairs 787 to 1026). We then excised the hybrid CSP gene from pIC-CSPfCT as a *Kpn*I-*Xho*I fragment and inserted it into the transfection plasmid, pR-CSPfCT. *Kpn*I and *Sac*I were used to release the inserted fragment from pR-CSPfCT prior to transfection of WT *P*. *berghei* (ANKA strain) parasites, as previously described [24]. Transgenic parasites were selected in Swiss Webster mice (NCI, Frederick, MD) by treatment with pyrimethamine (MP Biomedicals, Solon, OH) in drinking water (0.07 mg/ml). Pyrimethamine-resistant parasites were then cloned by limiting-dilution. Successful recombination at the 5’ and 3’ ends of the locus was verified by PCR. The primers used to confirm 5’ integration were 5’-F (TCACCCTCAAGTTGGGTAAAA) and PbPfCT-R (GCAGAGCCAGGCTTTATTCT); the primers to verify integration at the 3’ end were 3’-F (TGTAAAAATGTGTATGTTGTGTGC) and 3’-R (GTGCCCATTACGACTTTGCT). To verify that the cloned parasite population did not have contaminating WT *P*. *berghei* parasites, we developed a PCR assay using primers that flank the *SexA*I restriction site and then digested the resulting product with this enzyme. This restriction site is not present in the WT *P*. *berghei* CSP sequence but was inserted by replacement with our synthetic construct. The primers used for this PCR analysis were PbWT NT-F (TGTTACAATGAAGGAAATGATAATAAATTGTAT) and Pb 3’UTR-R (TCTTTTGGACATATATTCATTTTAGCA). Lastly, DNA isolated from the cloned chimeric parasites was sequenced to confirm the replacement of the *P*. *berghei* C-terminal region with the *P*. *falciparum* CSP C-terminus sequence. The sequence of the hybrid CS protein is provided in S1B Fig.

### *In vivo* protection assays

To measure liver parasite load, C57Bl/6 mice were challenged i.v. with 10,000 Pb/Pf or Pb/Pv hybrid sporozoites. Forty-eight hours later livers were harvested to assess the parasite load by RT-PCR as previously described [25]. We assessed sterile protection by monitoring the mice for development of blood-stage parasites after feeding by infected *Anopheles stephensi* mosquitoes. Briefly, prior to challenging mice, the percentage of infected mosquitoes was determined by choosing at least 10 mosquitoes from the pool and examining each salivary gland for the presence of sporozoites. Based on this information, the number of sporozoites used for the challenge was determined. The mice were anesthetized by i.p. injection of 250 μl of 2% avertin prior to feeding Pb/Pf- or Pb/Pv-infected *A*. *stephensi* mosquitoes for five minutes. After feeding, all mosquitoes were examined for the presence of blood in their gut to determine the number that took a blood meal. Daily blood smears were performed starting at 4 days after challenge. For measuring protection mediated by antibodies, Pb/Pf (described as CS(Pf) in [16]) or Pb/Pv [17] hybrid sporozoites were used for the challenge when VLPs targeting *P*. *falciparum* and *P*. *vivax* epitopes, respectively, were used as immunogens. For assessing protective efficacy of T cell epitopes, the new Pb-Pf-CSP-CT hybrid sporozoites described above were used for the challenge.

## Results

### Immunogenicity of VLPs carrying repeat versus non-repeat CS B cell epitopes

A number of interesting candidate epitopes outside the CS repeat domain have been described. For example, non-repeat CS B cell epitopes which have been shown to elicit *in vitro* neutralizing antibodies include: aa93-113 (lysine (K)-rich region), aa112-123 (conserved N1), and aa298-315 [26–28]. Similarly, a high percentage of adults and lesser numbers of children living in malaria endemic areas possess antibodies specific for CS C-terminal sequences that represent CD4^+^ and CD8^+^ recognition sites for human and murine T cells (i.e., UTC, TH3.R and CS.T3 regions) [29]. For several reasons the consideration of these non-repeat, CS B cell epitopes for vaccine design has been marginalized. Firstly, the immunodominance of the NANP and NVDP repeats and the established neutralizing efficacy of anti-CS repeat antibodies has reduced interest in non-repeat B cell epitopes somewhat [30–32]. Secondly, the induction of high titer CS-specific antibodies to non-repeat epitopes has been difficult with most immunogens. Our WHcAg platform technology allows insertion of virtually any CS sequence onto WHcAg. The resulting immunogens elicit high titer antibody even if the CS sequence is cryptic on the native CS protein. The Pb/Pf sporozoite technology allows evaluation of efficacy of these candidate vaccines by inserting the Pf B and T cell candidate epitopes in the CS protein of Pb sporozoites. The combination of these technologies permitted us to overcome the problems that have prevented analysis of the protective efficacy of CS non-repeat B and T cell sites in the past.

**Fig 1:**
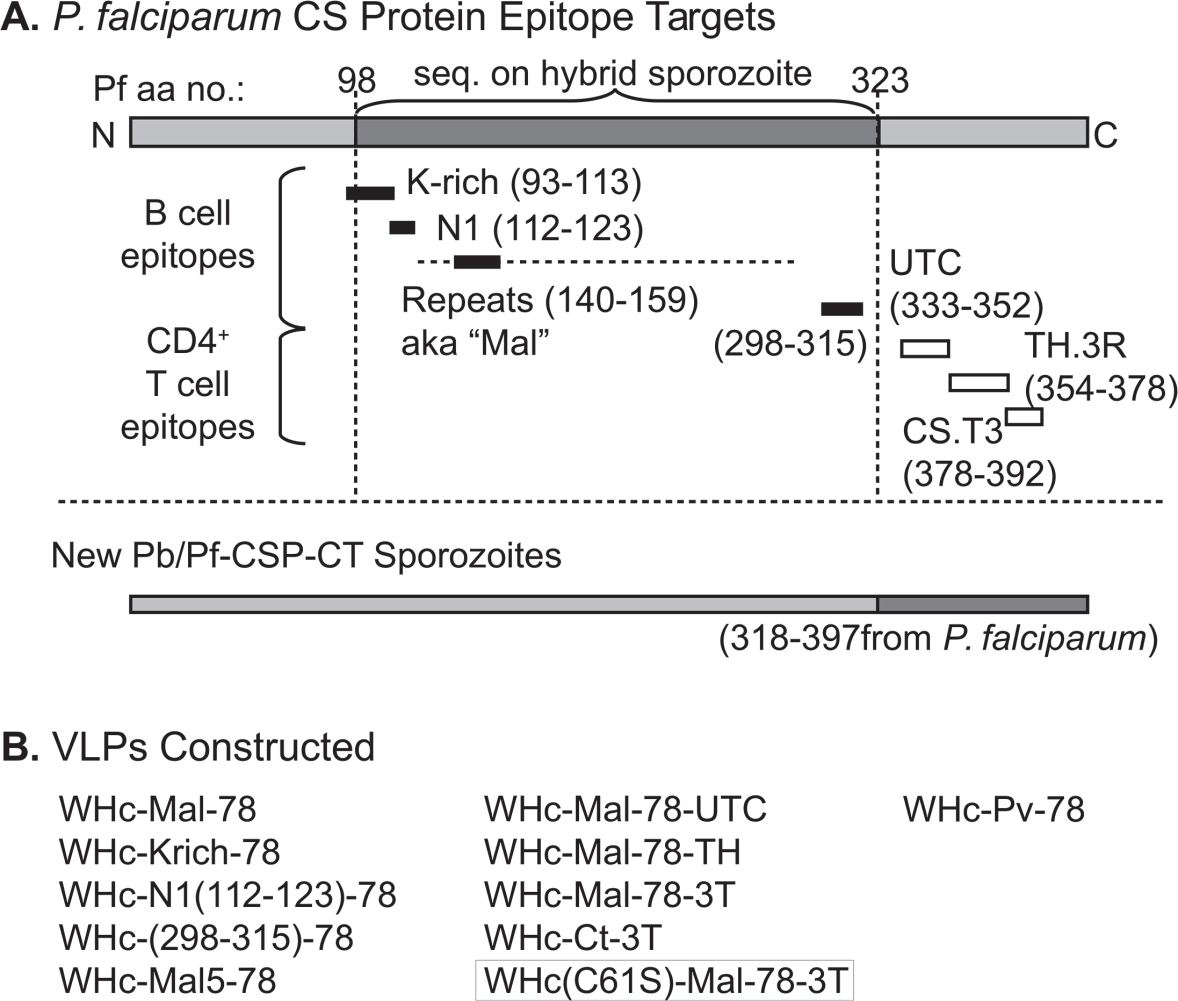
Targeted T and B cell epitopes on CS protein. (**A**) Schematic representation of the existing (upper panel) and novel (lower panel) hybrid Pb/Pf sporozoites. Native *P*. *berghei* sequence is indicated by light shading and transgenic *P*. *falciparum* sequence by dark shading. Neutralizing or presumptive neutralizing B-cell epitopes are denoted by black bars and human and murine CD4^+^ T cell epitopes by white bars. The “repeats” epitope (140–159) is part of a much larger motif, delineated by the dotted line. Numbering is based on the amino acid sequence of CSP from the 7G8 clone. (**B**) VLPs described in the manuscript. S2 Fig presents the amino acid sequences of the epitopes. In summary, Mal: NANP and NVDP repeats; Mal5: NANP repeat only; Ct: carboxy-terminal; TH: TH.3R and UTC epitopes; 3T: insertion of all 3 T cell eptiopes; C61S: Cys-to-Ser mutation at position 61 of WHcAg; Pv: *P*. *vivax* B cell repeats. Each of the epitopes is genetically inserted into the sequence of the VLP gene and a fully-assembled VLP consists of 120 homodimers, meaning each VLP presents 240 copies of the inserted epitope(s).

We produced, characterized and examined the immunogenicity of hybrid-WHcAg VLPs carrying the NANP/NVDP repeat epitopes and three selected non-repeat CS-specific B cell epitopes: the N1 region (aa 112–123); the K-rich region (aa 93–113); and the aa 298–315 region (see Fig 1) in comparison to full length rCS protein. Although the T cell response to CS protein is highly genetically restricted in mice and humans [33,34], we avoided this problem by immunizing a high responder strain (H-2^b^). Immunization with the full length rCS protein elicited very high antibody production to the 2 repeat epitopes, NANP and NVDP. However, consistent with a cryptic nature of the non-repeat CS B cell epitopes, immunization with rCS protein elicited no antibody to the CS_298-315_ region, extremely low antibody production to the N1 region (i.e., 1:1000 titer) and relatively low antibody production to the K-rich region (i.e., 1:125,000 titer) after primary and secondary immunization (Table 1). In contrast, both repeat and non-repeat B cell regions “excised” from the CS protein and inserted onto hybrid WHcAg VLPs elicited high levels of anti-insert antibodies (i.e., at least 1:3x10^6^ titers) (Table 1). Furthermore, the repeat and non-repeat anti-insert antibodies bound rCS protein in ELISAs. The repeat and non-repeat anti-insert antibodies also bound dry, hybrid Pb/Pf sporozoites to varying degrees as demonstrated by immunofluorescence assays (IFA) (Table 1). Interestingly, only the repeat-specific anti-insert antibodies (i.e., NANP/NVDP-specific) bound live sporozoites. These observations suggest that the three non-repeat B cell epitopes on the CS protein may be cryptic on intact, viable sporozoites.

**Table 1:** Characterization of WHc-CS VLPs carrying repeat and non-repeat B cell epitopes.

	endpoint dilution titers									
WHc-CS VLP (Insert)	α-WHc	α-NANP	α-NVDP	α-N1	α-K rich	α-CS (298–315)	α-CSP solid phase	IFA dry hybrid spzt	IFA viable hybrid spzt	Reduction in Liver Stage *in vivo*
**NANP/NVDP**	6 x 10^6^	15 x 10^6^	6 x 10^6^	–	–	–	>15 x 10^6^	16,000	++ >300	98%
**N1 (112–123)**	3 x 10^6^	–	–	3 x 10^6^	–	–	6 x 10^6^	1,800	–	18%
**K rich (93–113)**	625,000	–	–	–	3 x 10^6^	–	6 x 10^6^	600	–	0%
**CS (298–315)**	6 x 10^6^	–	–	–	–	3 x 10^6^	>15 x 10^6^	16,000	–	44%
**CS Protein** Full Length	0	6 x 10^6^	625,000	1,000	125,000	0	6 x 10^6^	ND	ND	ND

The listed WHcAg hybrid VLPs and full length rCS protein were used to immunize mice (2 doses; 20 μg and 10 μg in IFAd). Secondary antisera were pooled, serially diluted and analyzed by ELISA for binding to: solid phase WHcAg; repeat peptides (NANP)_5_ and (DPNANPNV)_2_; non-repeat peptides N1, Krich, CSP298-315; and rCS. Endpoint dilution titers are shown. Antisera were also evaluated by IFA on dry or viable sporozoites. The protective efficiency after *in vivo* challenge with 10,000 Pb/Pf sporozoites of mice immunized with the listed WHc-CS VLPs is also shown.

### Protective efficacy of VLPs carrying repeat versus non-repeat CS B cell epitopes

**Fig 2:**
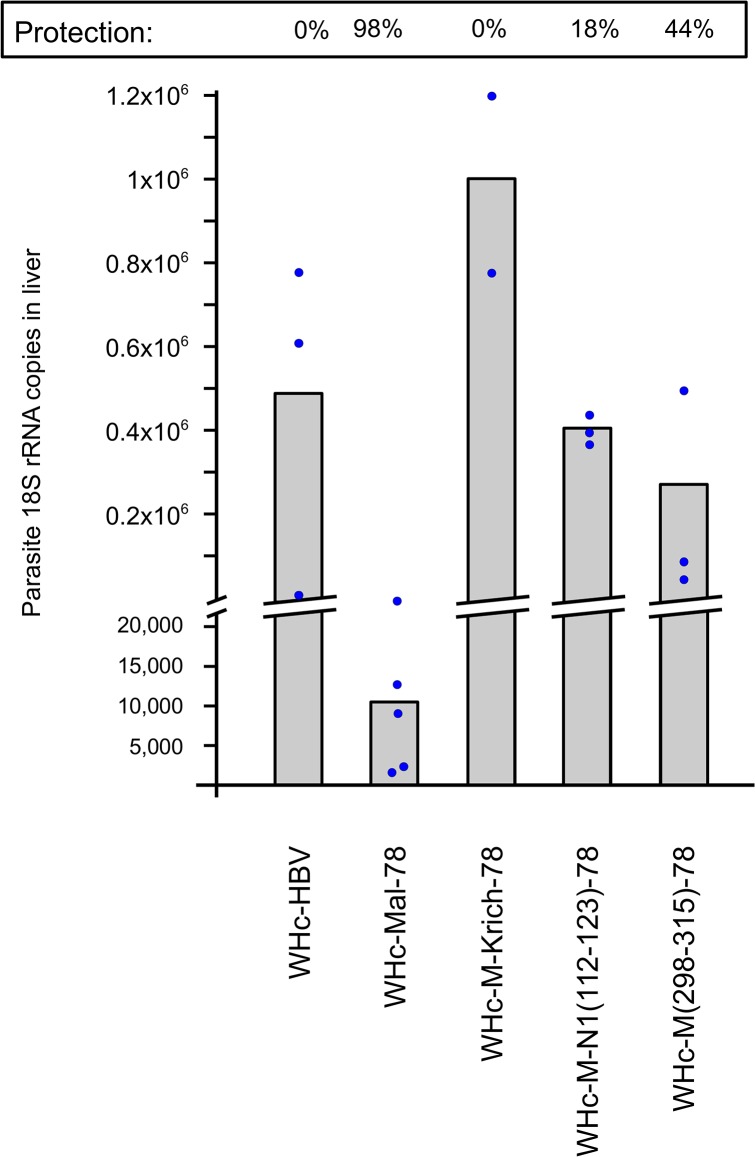
Comparison of protective efficacy of WHc-CS VLPs. Groups of mice were immunized with WHc-HBV negative control and CS-repeat (WHc-Mal-78) VLPs (2 doses of 20 μg and 10 μg in IFAd): CS-non-repeat VLPs (3 doses of 20 μg, 10 μg, 10 μg in IFAd). From 2 to 3.5 months after the last immunization dose all mice were challenged with 10,000 Pb/Pf sporozoites. Parasite 18S rRNA copy number in the liver was determined by qPCR 40 hours after infection. Circles represent individual mice and the bars represent mean values. % protection is based on mean values in comparison to the WHc-HBV negative control.

We also performed immunization/challenge experiments to determine the protective efficacy of hybrid WHcAg VLPs carrying the 2 repeat B cell epitopes (NANP/NVDP) and the three non-repeat B cell epitopes described above. As shown in Fig 2, immunization (2 doses of 20 and 10 μg) with VLPs carrying the repeat B cell epitopes protected mice challenged with 10,000 Pb/Pf sporozoites at a level of 98% in terms of parasite 18S rRNA copies detected in liver compared to mice immunized with a control hybrid WHcAg VLP carrying an irrelevant insert from the hepatitis B virus (HBV). In contrast, immunization (3 doses of 20, 10, and 10 μg) with the hybrid WHcAg VLPs carrying each of the three non-repeat B cell epitopes provided little to no protection (0–44%) against Pb/Pf sporozoite challenge despite the fact that high levels of anti-insert antibodies were present in the immunized mice (Fig 2 and Table 1). These results suggest that the non-repeat B cell epitopes may be cryptic on viable sporozoites *in vivo*. The results also suggest that it may not be productive to include these three non-repeat B cell epitopes in a CS-VLP vaccine candidate. A caveat to this interpretation is that the non-repeat B cell epitopes in the context of the VLPs may not represent the epitope structures present within the native CS protein, although anti-non-repeat Abs do bind rCS and dry sporozoites.

### Confirmation that CS repeat antibodies are predominant in providing protection

As an alternate approach to addressing the question of the importance of repeat vs. non-repeat CS-specific antibodies, we performed an experiment using rCS as the immunogen rather than hybrid VLPs. Mice were immunized with 2 doses of rCS (20 μg/10 μg) and the resulting antisera were pooled and pre-incubated with the 10,000 Pb/Pf sporozoites used for the challenge. This antisera provided significant protection compared to sporozoites pre-incubated in normal mouse sera (NMS). However, if the anti-rCS antisera was pre-adsorbed with repeat-containing VLPs (Δ NANP, NVDP) prior to being added to the 10,000 sporozoites, the protective efficacy was largely lost (S3 Fig).

### WHc-Mal-78-UTC elicits protective Abs in rabbits

**Fig 3:**
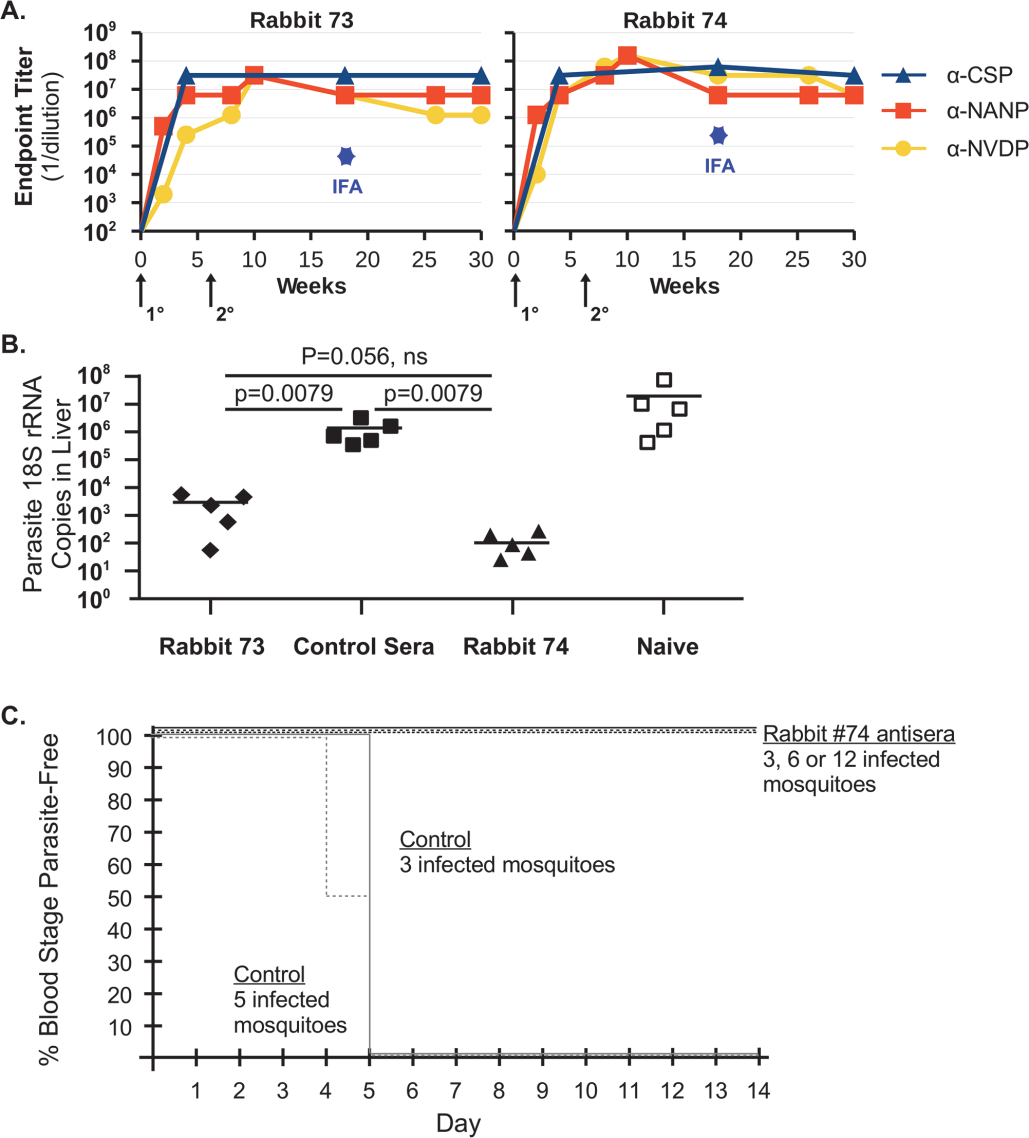
Immunogenicity of WHc-Mal-78-UTC in rabbits and protective efficacy of anti-VLP sera. (**A**) Animals were primed with 200 μg of WHc-Mal-78-UTC emulsified in IFAd and boosted at week 6 with 100 μg emulsified in IFAd (rabbit 73) or 200 μg in saline (rabbit 74). Serum was collected at the indicated time points and endpoint titers against NANP, NVDP and rCSP determined by ELISA. The sporozoite-specific IFA assay was performed on 18 week antisera and is represented by a star-shaped point. (**B**) Protection against liver stage Pb/Pf infection. Mice were injected with 500 μl of indicated rabbit antisera and challenged with 10,000 sporozoites i.v. shortly after receiving the antisera. Liver burden was determined by qPCR 40 hours after challenge. Control, normal mouse sera. P values are for Mann-Whitney U-test comparing in each case the 5 mice per group, ns = not significant at the 0.05 significance level. (**C**) Protection against blood stage Pb/Pf infection. 200 μl of sera from rabbit #74 or from a control naïve rabbit were passively transferred to groups of 7 or 4 mice, respectively, by i.v. injection. Mice were then challenged by allowing 3, 5, 6 or 12 mosquitoes infected with Pb/Pf sporozoites to feed on the mice for 5 min. Mice were bled daily starting on day 4 post-challenge and blood-stage infection assessed by microscopy on stained blood smears.

To examine protective efficacy of antisera from a second species, two rabbits were immunized with a VLP carrying the 2 CS repeat B cell epitopes and a malaria-specific human T cell epitope (UTC), designated WHc-Mal-78-UTC (Fig 3A). The antisera were passively transferred into naïve murine recipients. The recipients of anti-VLP rabbit sera were either challenged intravenously (i.v.) with 10,000 Pb/Pf sporozoites and parasite burden in the liver determined (Fig 3B) or challenged by the bites of infected mosquitoes and blood-stage parasitemia monitored over a 10–14 day period (Fig 3C). As shown in Fig 3, both rabbits (#73 and #74) produced high titer anti-NANP, anti-NVDP and anti-rCS Abs detected by ELISA and by IFA on hybrid sporozoites (Fig 3A). Antisera (0.5 ml) from both rabbits were passively transferred (i.v.) to naïve mice and the mice were immediately challenged with 10,000 Pb/Pf sporozoites (i.v.). Forty hours later the parasite liver burdens were determined. Passively transferred anti-VLP sera from both rabbits significantly reduced the parasite liver burden as compared to control rabbit sera, although rabbit #74 sera was most effective (Fig 3B). It is notable that rabbit #74 was primed with WHc-Mal-78-UTC emulsified in IFAd but boosted with the VLP in saline, whereas, rabbit #73 was primed and boosted in IFAd. This suggests that there may be no advantage to the use of potent adjuvants after the primary injection of the VLPs. Rabbit #74 serum was chosen to passively transfer (0.2 ml) to murine recipients, which were challenged with the bites of from 3 to 12 Pb/Pf-infected mosquitoes over a five minute time frame. Blood stage parasitemia was monitored for the next 10–14 days. All 21 mice receiving the anti-WHc-Mal-78-UTC rabbit sera were totally protected from blood stage parasitemia regardless of exposure to 3, 6 or 12 infected mosquitoes. The 8 control mice exposed to 3 or 6 infected mosquitoes demonstrated infection by day 4 or 5 (Fig 3C). However, it is interesting to note that the adoptive transfer of 0.2 ml of a 1:3 dilution of rabbit #74 sera failed to protect mice against blood stage infection. These studies demonstrate that the protective efficacy elicited by WHc-Mal-78-UTC VLPs can be mediated solely by anti-CS repeat Abs, but a threshold level of protective Abs is required.

### Addition of CS-specific T cell domains

**Fig 4:**
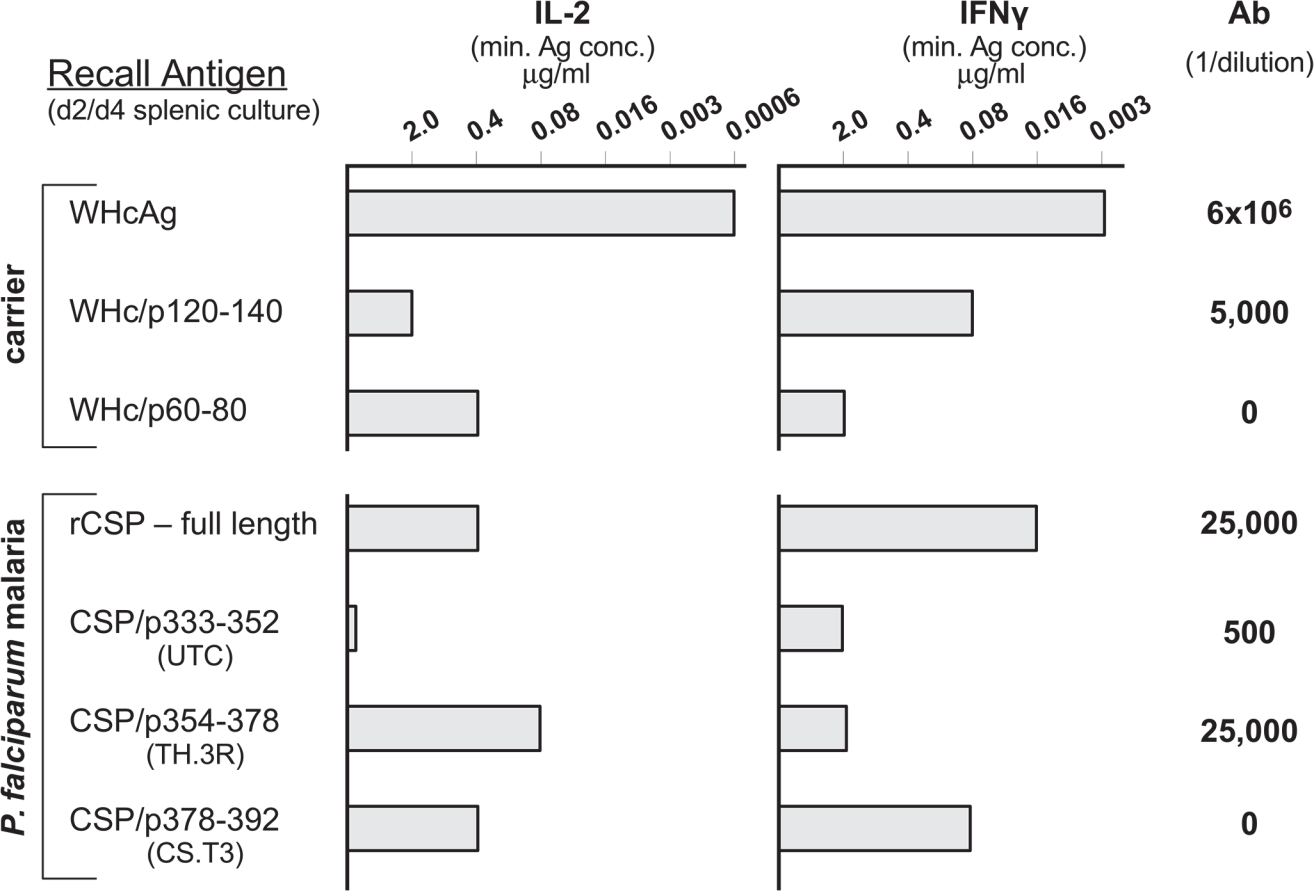
WHc-Ct-3T primes malaria-specific as well as WHcAg-specific CD4^+^ T cells. To assess T cell priming, three mice were immunized with WHc-Ct-3T (a single 20 μg dose in IFAd) and 10 days later spleen cells were harvested and cultured as pools with varying concentrations of the indicated recall antigens. Culture supernatants were collected at day 2 for determination of IL-2 and day 4 for determination of IFNγ. The minimum concentration of each antigen necessary to yield detectable cytokine is shown. For antibody production, mice were immunized (20 μg/IFAd) and boosted (10 μg/IFAd).

An important goal is to add CS-specific T cell sites to vaccine candidates in order to prime CS-specific CD4^+^/CD8^+^ T cells as well as elicit CS-specific neutralizing antibodies. For this purpose, we added 1, 2 or all 3 (i.e., UTC, TH.3R, and CS.T3) well characterized human T cell domains to a standard hybrid WHcAg VLP carrying the 2 CS specific repeats (i.e. WHc-Mal-78). The T cell domains were added to the C-terminus of the hybrid WHcAg VLPs and all 3 hybrid VLPs were successfully produced and were shown to be approximately equally immunogenic in terms of anti-NANP and anti-NVDP antibody production (S4 Fig). In order to determine the contribution of CS-specific T cells to the protective efficacy of candidate VLP vaccines, the established protective efficacy of anti-NANP/NVDP antibodies had to be excluded. For that purpose, we constructed a hybrid WHcAg VLP carrying only the 3 T cell regions and devoid of the neutralizing CS repeat B cell epitopes designated WHc-Ct-3T. As shown in Fig 4, immunization with WHc-Ct-3T primed both WHcAg-specific and CS protein-specific CD4^+^ T cells as determined by cytokine production elicited by splenic T cells cultured with a panel of WHcAg and CS protein-specific proteins and peptides. Also note that WHc-Ct-3T immunization elicited low level Ab production to rCS and the TH.3R site, which is also a B cell epitope in addition to a CD4^+^ T cell epitope. Because the Pb/Pf hybrid sporozoites used in the previous studies do not contain the *P*. *falciparum* T cell domains, a new transgenic Pb sporozoite (Pb/Pf-CSP-CT) containing the complete C-terminus (i.e., aa318-397) from the Pf CS protein was produced (Fig 1; S1 Fig). Therefore, we were able to perform an immunization/challenge experiment with WHc-Ct-3T VLPs. Although WHc-Ct-3T was immunogenic for both CS-specific B and CD4^+^ T cell epitopes (Fig 4), no protection against a 10,000 Pb/Pf-CSP-CT sporozoite challenge was elicited.

### Effect of the 3 CS-specific T cell domains on protective efficacy

**Fig 5:**
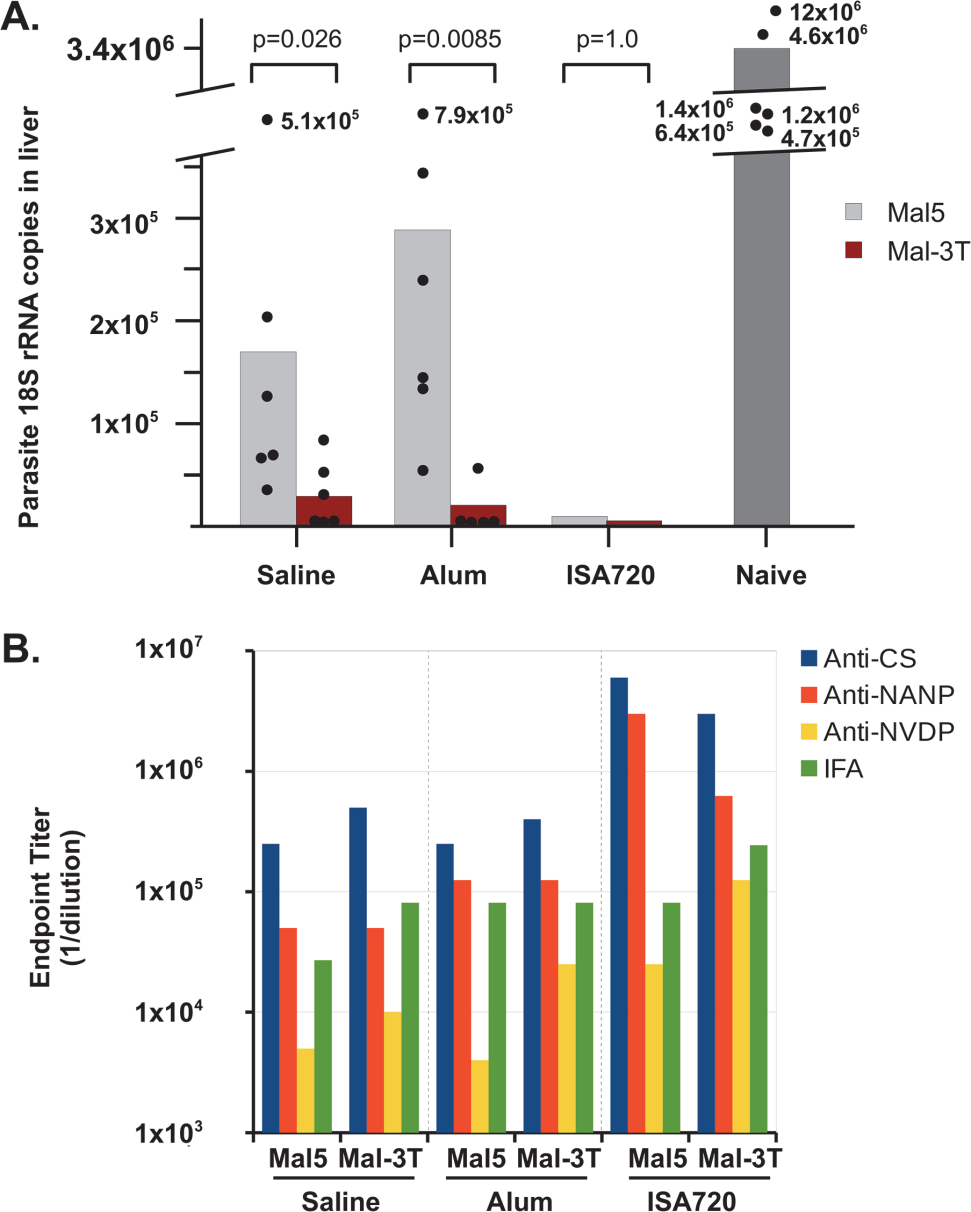
Comparison of protective efficacy and serology for WHc-Mal5-78 (Mal5) vs WHc-Mal-78-3T (Mal-3T). Groups of 6 mice each were immunized with WHc-Mal5-78 or WHc-Mal-78-3T formulated in either saline, alum or Montanide ISA-720 and given a single booster injection. After the boost mice were challenged with 10,000 hybrid sporozoites. (**A**) Liver burden was assessed by determining parasite 18S rRNA copies in the liver. Circles represent individual mice, boxes represent mean values. P values are for Mann-Whitey *U*-test between groups for each formulation, with each group having six mice, except the Mal-3T in alum group, which had five mice. (**B**) Post-boost antibody levels in pooled sera were determined by ELISA using rCSP, NANP or NVDP peptides as solid phase ligands. IFA titers were performed on dry Pb/Pf sporozoites.

Because anti-NANP-specific Abs play a dominant role in protection, we compared the protective efficacy of a standard VLP (WHc-Mal5-78) containing only the 4 NANP repeats, which was previously shown to elicit significant protection against a Pb/Pf sporozoite challenge, with a VLP containing the NANPNVDP(NANP)_3_ B cell insert in the loop of WHcAg plus all 3 T cell domains (WHc-Mal-78-3T) inserted at the C-terminus of WHcAg. Groups of 6 mice were primed and boosted with WHc-Mal5-78 or WHc-Mal-78-3T formulated either in saline only (200 μg VLPs), alum (100 μg VLPs) or Montanide ISA720 (50 μg VLPs) (Fig 5). Both VLPs elicited significant reduction in parasite liver burden (at least 90% reduction in parasite 18S rRNA copies in liver) in all three formulations compared to naïve challenged control mice (Fig 5A). However, the VLP carrying the three T cell domains (WHc-Mal-78-3T) elicited statistically superior protection in saline (99.1% vs 95% protection) and in alum (99.2% vs 91.7% protection) compared to the (NANP)_4_ B cell only-containing VLP (WHc-Mal5-78). Both VLPs were equally protective when formulated in Montanide ISA720 (Fig 5A). Anti-CS, anti-NANP, and anti-NVDP antibodies were measured by ELISA and IFAs were performed to determine if differential antibody levels would explain the superior protective efficacy of the WHc-Mal-78-3T VLP formulated in saline and alum (Fig 5B). No significant serological differences were noted between the two VLPs. However, there was a trend towards higher titer anti-NVDP repeat antibodies in the WHc-Mal-78-3T-immunized groups, especially when formulated in alum. This was expected because the WHc-Mal5 VLP does not contain the NVDP repeat. However, the polyclonal anti-NANP antibodies elicited by the WHc-Mal5 VLP demonstrated cross-reactivity for the (NVDP)_2_ peptide. IgG isotype testing also revealed no significant differences between anti-CS antibodies elicited by WHc-Mal5 and WHc-Mal-78-3T VLPs. This suggests that malaria-specific CD4^+^ T cells primed by immunization with the WHc-Mal-78-3T VLP may have contributed to the greater efficacy either indirectly by providing an additional source of T helper cell function or, more likely, by directly exerting a negative effect on liver stage development via cytokine production. Although the hybrid sporozoites used for challenge did not contain the Pf T cell domains engineered into the WHc-Mal-78-3T VLPs, the 3 T cell domains of *P*. *falciparum* and *P*. *berghei* share a significant degree of homology as shown in S5 Fig. In any event, the superior performance of the WHc-Mal-78-3T VLP elevated this VLP to a primary vaccine candidate.

### A WHcAg-CS VLP in alum elicits sterile immunity to blood stage malaria

WHc-Mal-78-3T performed well in terms of reducing parasite load in the liver after a 10,000 Pb/Pf sporozoite challenge (up to 99.98% reduction, Fig 5), however, to determine if this level of reduction in liver burden is sufficient to yield full protection from blood stage parasitemia an immunization/challenge experiment monitoring blood stage parasitemia as the final endpoint is required because a single surviving sporozoite infecting the liver can result in a blood stage infection [35]. For this experiment we modified WHc-Mal-78-3T by a point mutation (C61S) in the WHcAg, which eliminated the intermolecular disulfide bond at residue 61. The C61S mutation in WHcAg-hybrid VLPs was chosen because it can reduce anti-WHc (carrier-specific) antibody production and/or increase anti-insert antibody production.

**Fig 6:**
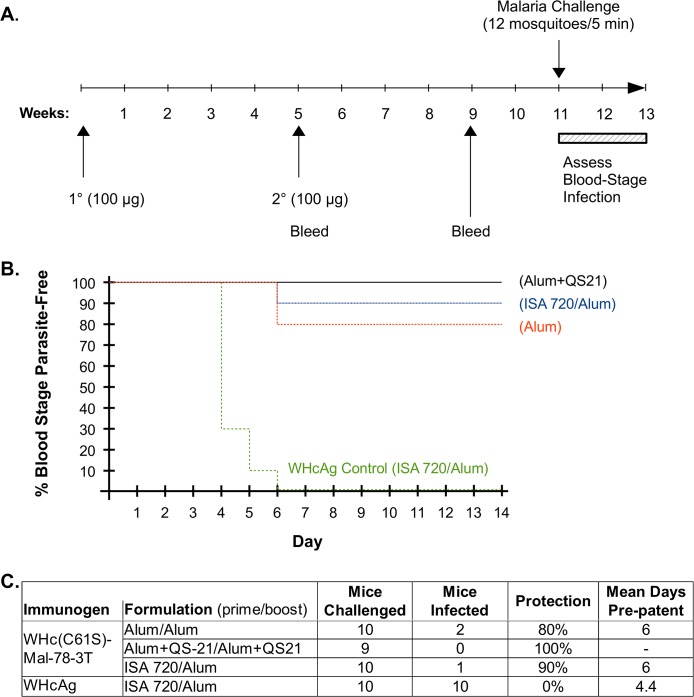
Protective efficacy of WHc(C61S)-Mal-78-3T immunization against *P*. *berghei*/*P*. *falciparum* blood stage malaria infection. Groups of ten mice were primed and boosted with 100 μg of WHc(C61S)-Mal-78-3T formulated in alum (250 μg/dose), alum + QS21 (20 μg/dose) or emulsified in Montanide ISA720 (50% vol/vol) and boosted in alum as indicated. (**A**) Time line showing schedule of prime, boost and challenge with 12 mosquitoes infected with Pb/Pf hybrid sporozoites allowed to feed on the mice for 5 min. After feeding, mosquitoes were examined for blood in the gut, confirming that multiple mosquitoes had fed on each animal. (**B**) Graphic representation of the percentage of mice remaining protected (i.e., free of blood stage parasites) during the 14 day monitoring period. (**C**) Tabular summary of results. One mouse from the Alum+QS21 group died before challenge.

Groups of 10 mice each were immunized and boosted with 100 μg of the WHc(C61S)-Mal-78-3T VLP either formulated in alum, alum+QS-21, or primed with an emulsion of Montanide ISA 720 (50%) and boosted in alum. The control group was primed with 100 μg of WHcAg (no insert) emulsified in Montanide ISA 720 and boosted in alum (Fig 6). Six weeks after the boost mice were challenged by exposure to the bites of 12 Pb/Pf-infected mosquitoes for 5 minutes. This method of challenge was chosen because it represents a more physiologically relevant route of infection as compared to i.v. injection of sporozoites. Blood was sampled over the next 14 days and examined for parasitemia. As shown in Fig 6, 10 of 10 WHcAg-immunized control mice became positive for blood stage malaria within a mean of 4.4 days. In contrast, 0 of 9 mice immunized with WHc(C61S)-Mal-78-3T formulated in alum+QS-21 became infected; 1 of 10 mice immunized in Montanide/alum became infected; and 2 of 10 mice immunized in alum became infected. The 3 of 29 mice in the experimental groups that did become infected demonstrated delayed parasitemia (mean of 6.0 days), suggesting a possible elimination of 99% of sporozoites given that 90% elimination is required to obtain a one day delay in developing a patent blood stage infection. The serology of each group pre-challenge and of the survivors three months post-challenge is shown in S1 Table. Although anti-CS Ab titers decreased over time, anti-CS Abs were still in excess of 1x10^6^ endpoint titers three months post-challenge in all adjuvant groups. The apparent lack of a boost to the anti-CS Ab titers may reflect the low immunogenicity of the protein in the context of the parasite infection.

### Preliminary evaluation of a VLP carrying P. vivax CS epitopes

**Fig 7:**
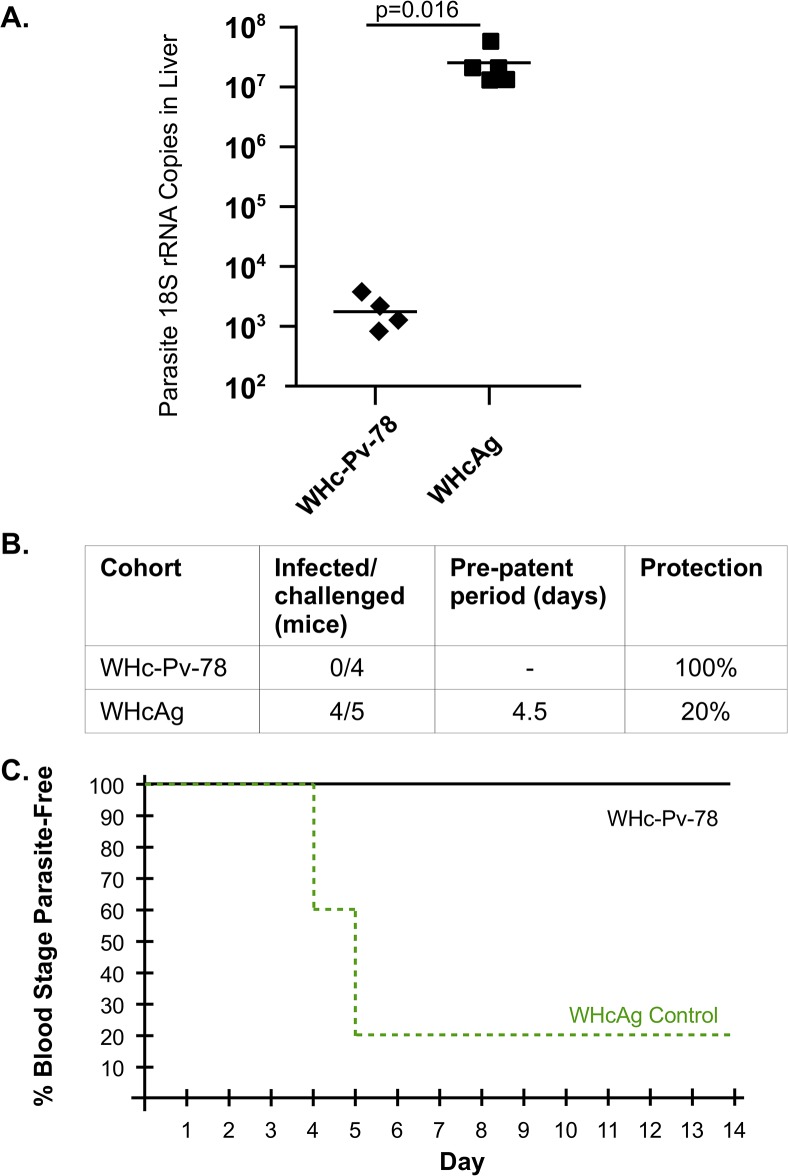
Protective efficacy of WHc-Pv-78 immunization against *P*. *berghei*/*P*. *vivax* malaria infection. Mice were primed and boosted with 100 μg of WHc-Pv-78 or WHcAg emulsified in IFAd (50% vol/vol). (**A**) Mice were challenged with 10,000 Pb/Pv sporozoites injected in the tail vein and liver infection determined by qPCR. P value is for the Mann-Whitney *U*-test with four mice in the WHc-Pv-78 group and five mice in the control WHc group. (**B & C**) Mice were exposed to the bites of 10 Pb/Pv-infected mosquitoes for 5 min, after which, mosquitoes were examined for blood in the gut to confirm that multiple mosquitoes had fed on each animal. Malarial infection was determined by blood smear during the 14 day monitoring period. Results are depicted in tabular (B) and graphic (C) forms.

A hybrid WHcAg VLP carrying 2 copies each of both variants of type 1 (VK210) CS repeat epitopes from *P*. *vivax* parasites was constructed (WHc-Pv-78). *In vivo* protective efficacy was evaluated using hybrid *P*. *berghei/P*. *vivax* (Pb/Pv) sporozoites expressing the repeat region of the *P*. *vivax* CS protein (both VK210 variants) [17]. Immunization with 2 doses of varying amounts of WHc-Pv-78 VLPs in either incomplete Freund's adjuvant (IFAd) or alum elicited high titer anti-CS PV repeat antibodies as detected on solid phase peptide and verified by IFA assay on Pb/Pv hybrid sporozoites (S2 Table). Immunization with 100 μg of WHc-Pv-78 in IFAd elicited extremely high titer anti-CS Pv repeat antibodies (1.5x10^8^). This immunization schedule was chosen to examine the protective efficacy of WHc-Pv-78 VLPs against experimental liver infection as well as blood stage infection with hybrid Pb/Pv sporozoites in mice. As shown in Fig 7A, immunization with WHc-Pv-78 VLPs provided 99% protection in terms of parasite 18S rRNA copies detected in the liver compared to mice immunized with the WHcAg carrier after challenge with 10,000 Pb/Pv sporozoites. To determine if this level of reduction in liver parasite burden was sufficient to provide sterile immunity to blood stage infection, WHc-Pv-78 VLP-immunized and WHcAg-immunized mice were challenged by exposure to the bites of 10 Pb/Pv-infected mosquitoes for 5 minutes. Whereas 4 of 5 control mice became infected in a pre-patent period of 4.5 days, 0 of 4 WHc-Pv-78-immunized mice were infected over an observation period of 14 days (Fig 7B).

## Discussion

An epitope-focused approach was utilized to present selected B and T cell epitopes from the CS protein of the *P*. *falciparum* malaria parasite on the heterologous WHcAg carrier platform. Two repeat and three non-repeat B cell epitopes from the CS protein were inserted into the WHcAg carrier. Although all hybrid VLPs elicited high levels of anti-insert Abs, only hybrid VLPs carrying the CS repeat B cell epitopes (NANP and NVDP) provided significant protection of the liver (98%) against an experimental challenge with hybrid Pb/Pf sporozoites in mice. Whereas, anti-CS repeat and anti-CS non-repeat Abs bound dry Pb/Pf sporozoites, only anti-CS repeat Abs bound viable sporozoites. This data suggests that the 3 non-repeat B cell epitopes are poorly expressed or cryptic on viable sporozoites. Addition of 3 well-defined malaria-specific human T cell domains to the hybrid VLPs enhanced protective efficacy in the liver as well as primed malaria-specific CD4^+^ T cell cytokine production. However, immunization with hybrid VLPs carrying only the 3 malaria-specific T cell domains was unable to provide protection, indicating that anti-CS repeat Abs are necessary for protection. In fact, anti-CS repeat Abs are sufficient for protection against liver stage as well as blood stage infection as demonstrated by sterile immunity to blood stage infection following adoptive transfer of rabbit anti-VLP antiserum. Furthermore, active immunization with a hybrid VLP, designated WHc(C61S)-Mal-78-3T, elicited sterile immunity to blood stage infection in 26 of 29 mice and delayed parasitemia in the remaining 3 mice, depending on adjuvant formulation. The alum + QS-21 formulation was the most efficient adjuvant and yielded 100% protection from blood stage infection. The results indicate that immunization with an epitope-focused VLP containing selected B and T cell eptiopes from the *P*. *falciparum* CS protein formulated in adjuvants acceptable for human use can elicit sterile immunity against blood stage malaria if sufficient anti-CS protective Abs are produced. The appropriate adjuvant formulation to achieve protective Ab levels in humans as well as VLP dose will need to be determined in clinical trial.

In previous studies we demonstrated that CS repeat B cell epitopes from both *P*. *berghei* and *P*. *yoelii* murine parasites inserted onto the HBcAg conferred 80–100% protection against blood stage infection in immunized mice [13,14]. We extrapolated that strategy to *P*. *falciparum* CS-derived B and T cell epitopes and produced HBcAg-CS hybrid VLPs [designated V12.PF3.1[15]/ICC1132[36]] that were highly immunogenic in rodents and non-human primates, respectively. Unfortunately, a flawed Phase IIa clinical trial, in which a suboptimal dose of ICC1132 (5 μg equivalent of CS repeat B cell epitope) given in a single injection without a boost, did not permit the realistic efficacy of this hybrid VLP to be determined in humans [37]. In this current study we developed a species variant of the HBcAg, the WHcAg, as a platform for *P*. *falciparum*/*P*. *vivax* CS epitopes in order to avoid the disadvantages of using a carrier derived from a human pathogen [38]. This is especially important for a malaria vaccine because HBV and malaria are co-endemic in many regions of the world and chronic HBV carriers are often immune tolerant to both HBcAg and HBsAg (note that the HBsAg is used as a carrier in the RTS,S vaccine). Additional modifications to the WHc(C61S)-Mal-78.3T vaccine candidate compared to ICC1132 are: the use of the full length WHcAg to accommodate the encapsidation of ssRNA as a TLR7 ligand, which enhances immunogenicity [39]; incorporation of additional malaria-specific T cell domains; and mutation of the WHcAg cysteine 61, which eliminates intermolecular disulfide bonds common to both WHcAg and HBcAg. The C61S mutation in hybrid VLPs can reduce anti-WHc (carrier-specific) Ab production and/or increase anti-insert Ab production. For these reasons the WHcAg is a superior choice to the HBcAg as a VLP platform for malaria CS epitopes. It would be useful to directly compare CS-based vaccine candidates, including the industry-standard RTS,S, in a standardized hybrid Pb/Pf challenge model as a preclinical selection tool. A number of CS-based vaccines have been developed recently [40–43]. Typically, protective efficacy has been determined using different challenge methods and different chimeric rodent parasites, making comparisons difficult. For example, hybrid Pb/Pf parasites used herein express an extended CS repeat region from the Pf CS protein [16], whereas, other hybrid Pb/Pf parasites used for challenge experiments [40,42] contain the full-length Pf CS protein [44].

In the absence of head-to-head comparative studies to date, the WHc(C61S)-Mal-78-3T candidate embodies a number of unique characteristics that may be advantageous in comparison to other CS-vaccine candidates. The enhanced immunogenicity and protective efficacy of WHc(C61S)-Mal-78-3T suggests that the suboptimal performance of a preerythrocytic vaccine candidate is not likely due to the selection of the CS repeat region as a target or to a paucity of B cell epitopes, but rather to insufficient production of protective Abs. For example, the RTS,S vaccine shares similar CS-specific B and T cell epitopes with WHc(C61S)-Mal-78-3T but the carrier moieties are markedly different. WHc(C61S)-Mal-78-3T efficiently self-assembles into hybrid VLPs, which are stable even at 65°C, whereas the HBsAg-based RTS,S requires the addition of excess native HBsAg particles. Compared to the HBsAg, hepadnavirus nucleocapsids are inherently more immunogenic in mice and humans during natural infection or after immunization [45–48], are less susceptible to MHC restricted non-responsiveness and can function as T cell-independent immunogens [45,48]. Finally, use of the WHcAg would circumvent HBV-specific immune tolerance present in populations endemic for HBV that are often endemic for malaria as well [38]. As a practical matter, WHcAg-CS VLPs are produced in high yields in bacteria and are extremely heat stable, therefore, production costs are relatively low and no cold-chain is required.

The immunogenicity and protective efficacy of WHcAg hybrid VLPs carrying *P*. *vivax* CS repeat B cell epitopes demonstrates the power and flexibility of the WHcAg VLP combinatorial technology, especially when combined with the hybrid Pb/Pv sporozoite technology [17] used for challenge experiments. In fact, WHc-Pv-78 VLPs represent the first example of a *P*. *vivax* immunogen capable of eliciting sterile immunity to blood stage infection in this hybrid Pb/Pv challenge model after active immunization. It is notable that passive transfer of 400 μg of the *P*. *vivax* CS-specific Mab 2F2 was not able to confer sterile immunitiy against a 5 min exposure to the bites of 4 Pb/Pv-infected mosquitoes [17]. The failure of Mab 2F2 to transfer sterile immunity demonstrates that Pb/Pv sporozoites are highly infectious and represent a stringent model to evaluate the protective efficacy of *P*. *vivax* CS-targeted immunogens such as WHc-Pv-78. The CS repeat epitopes and variants from other *P*. *vivax* strains can also be inserted onto WHcAg-CS hybrid VLPs. Further development of *P*. *falciparum* and *P*. *vivax*-specific WHcAg-CS hybrid VLPs would allow their use either separately or combined in a bivalent malaria vaccine, depending on the regional malaria threat.

## Supporting Information

S1 Fig Development and characterization of hybrid PB/PF parasites carrying the PF C-Terminus (PB/PF-CSP-CT). (**A**) Scheme representing the strategy used for replacing the CSP gene of *P*. *berghei* (ANKA) with the *P*. *falciparum* (3D7) C-terminal region. The annealing sites of the primers used to verify recombination by PCR are indicated below. Restriction sites shown are K—*Kpn*I; Se-*SexA*I; P-*Pac*I; Xh—*Xho*I; S—*Sac*I. (**B**) The resulting amino acid sequence of the hybrid CS protein. The sequence derived from the *P*. *falciparum* (3D7) sequence is depicted in red text.
(TIF)

S2 Fig CS Epitope Sequences. (**A**) Amino acid sequences of each epitope engineered onto the WHc VLPs (see Fig 1 for list of VLPs). (**B**) Amino acid sequences of the synthetic peptides used for antibody titer determination by ELISA.
(TIF)

S3 Fig Only anti-CS repeat antibodies protect against a sporozoite challenge. Mice were immunized with rCS and sera either unadsorbed or adsorbed with NANP/NVDP-containing VLPs (ΔNANP, NVDP) were incubated with 10,000 sporozoites prior to challenge. NMS, normal mouse sera.
(TIF)

S4 Fig Comparison of WHc-CS VLPs containing malaria-specific T cell epitopes. Groups of 3 mice were immunized (2 doses: 20 μg and 10 μg in IFAd) with the indicated WHcAg hybrid VLPs. Secondary antisera were pooled and serially diluted and analyzed by ELISA for binding to solid-phase WHcAg, NANP and NVDP. End-point titers of pooled sera are shown.
(TIF)

S5 Fig Conservation of T cell epitopes on *P*. *falciparum* and *P*. *berghei* CS. Alignment of the *P*. *berghei* UTC, TH.3R and CS.T3 T cell domains with the *P*. *falciparum* T cell domains incorporated into the WHc-Mal-78-3T VLP. The percentage represents homologies between the two sequences.
(TIF)

S1 Table Kinetics of IgG Ab titers through primary immunization (1°) with WHc(C61S)-Mal-78-3T, at the boost (2°) and at 3 months post-challenge. Mean endpoint dilution titers from 9–10 mice in each group are shown.
(PDF)

S2 Table Immunogenicity of WHc-Pv-78 VLPs. Mean endpoint dilution Ab titers shown. 1°, primary; 2°, secondary antisera. IFA assay used Pb/Pv dry sporozoites.
(PDF)
