# Supplementary figures and images for: P. falciparum and P. vivax Epitope-Focused VLPs Elicit Sterile Immunity to Blood Stage Infections
Source: PLoS One. 2015 May 1;10(5):e0124856. doi: 10.1371/journal.pone.0124856 (PMC4416889; doi:10.1371/journal.pone.0124856)

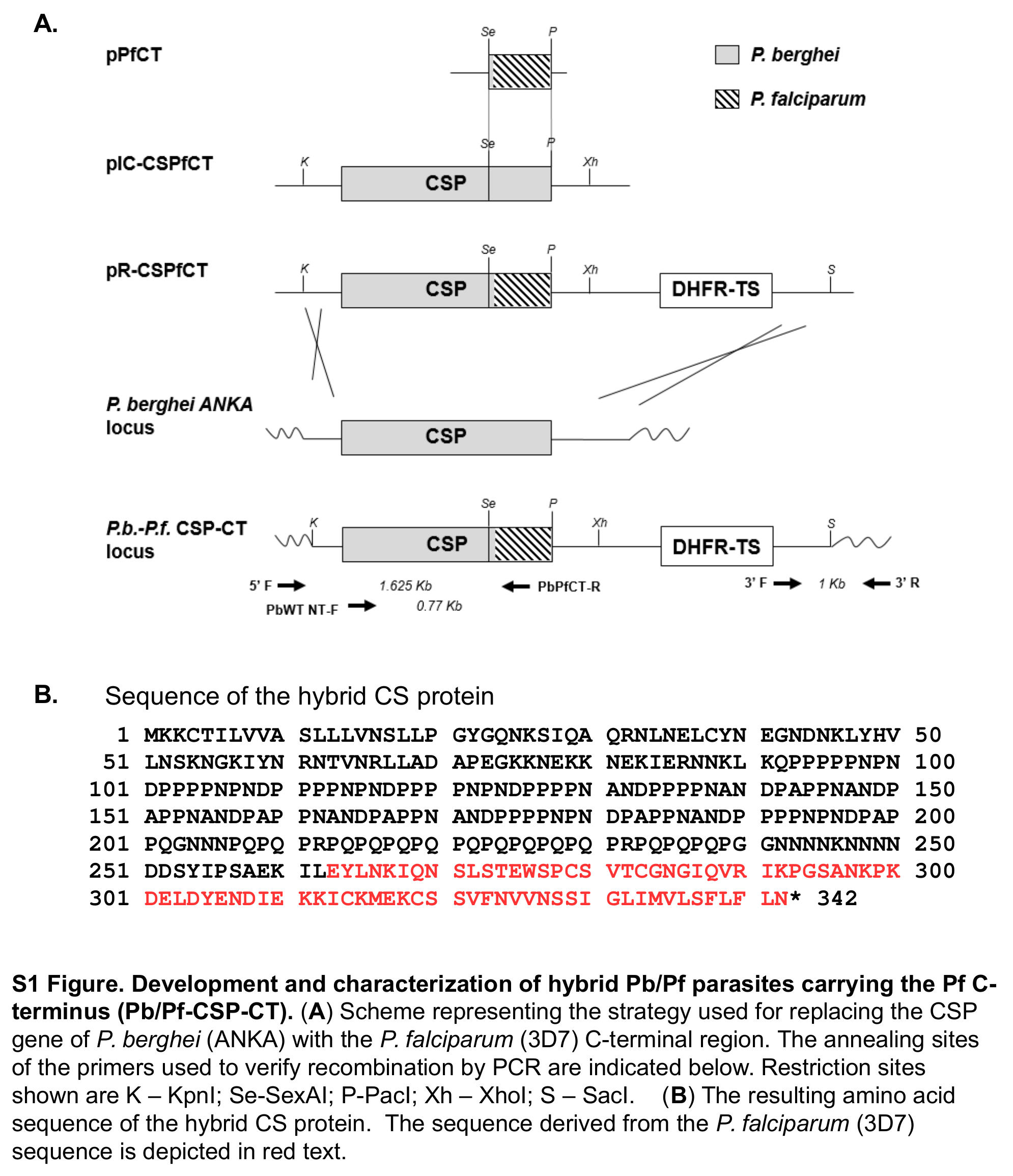

Supplement: S1 Fig — (A) Scheme representing the strategy used for replacing the CSP gene of P. berghei (ANKA) with the P. falciparum (3D7) C-terminal region. The annealing sites of the primers used to verify recombination by PCR are indicated below. Restriction sites shown are K—KpnI; Se-SexAI; P-PacI; Xh—XhoI; S—SacI. (B) The resulting amino acid sequence of the hybrid CS protein. The sequence derived from the P. falciparum (3D7) sequence is depicted in red text. (TIF) [file pone.0124856.s001.tif]

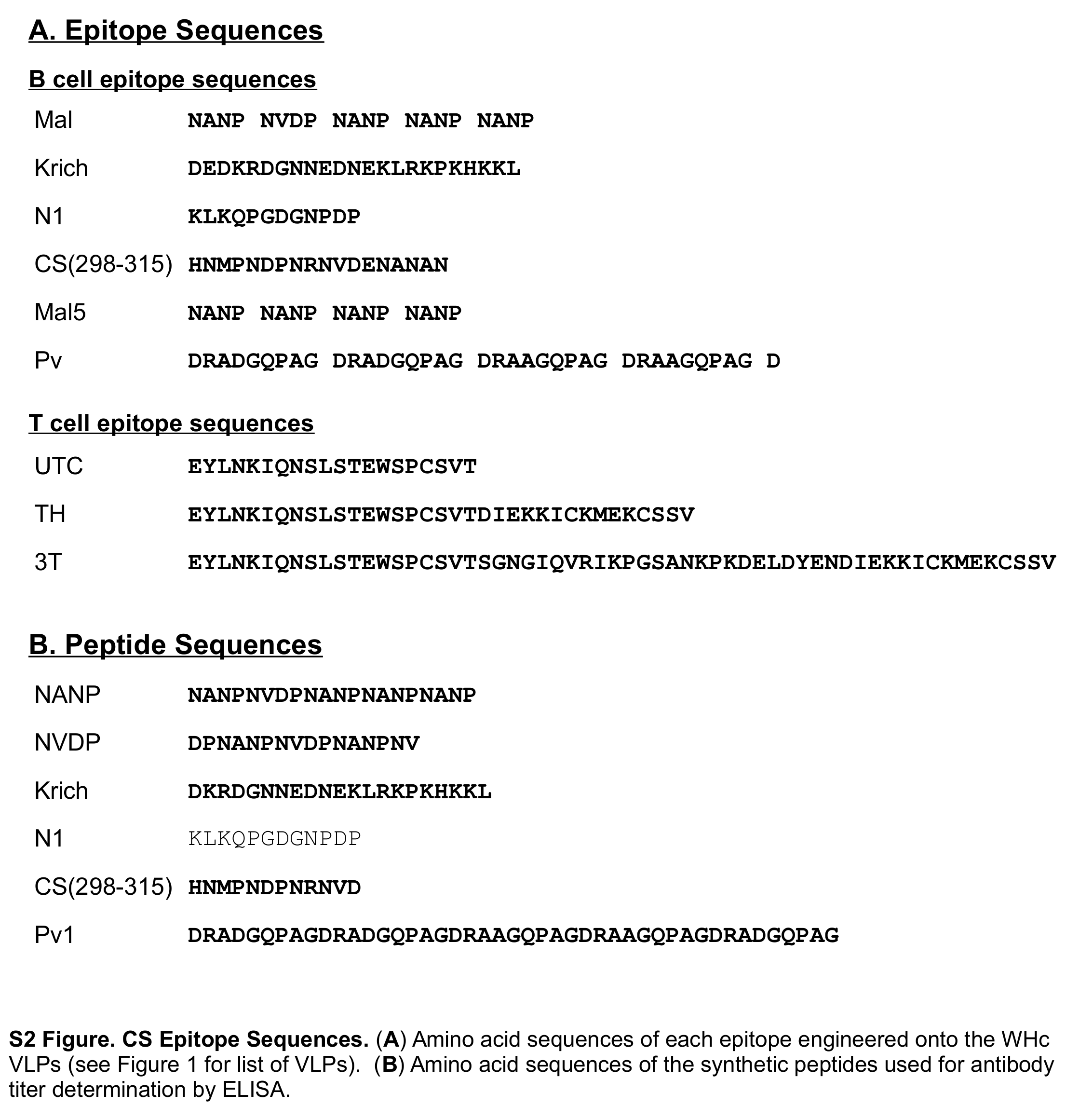

Supplement: S2 Fig — (A) Amino acid sequences of each epitope engineered onto the WHc VLPs (see Fig 1 for list of VLPs). (B) Amino acid sequences of the synthetic peptides used for antibody titer determination by ELISA. (TIF) [file pone.0124856.s002.tif]

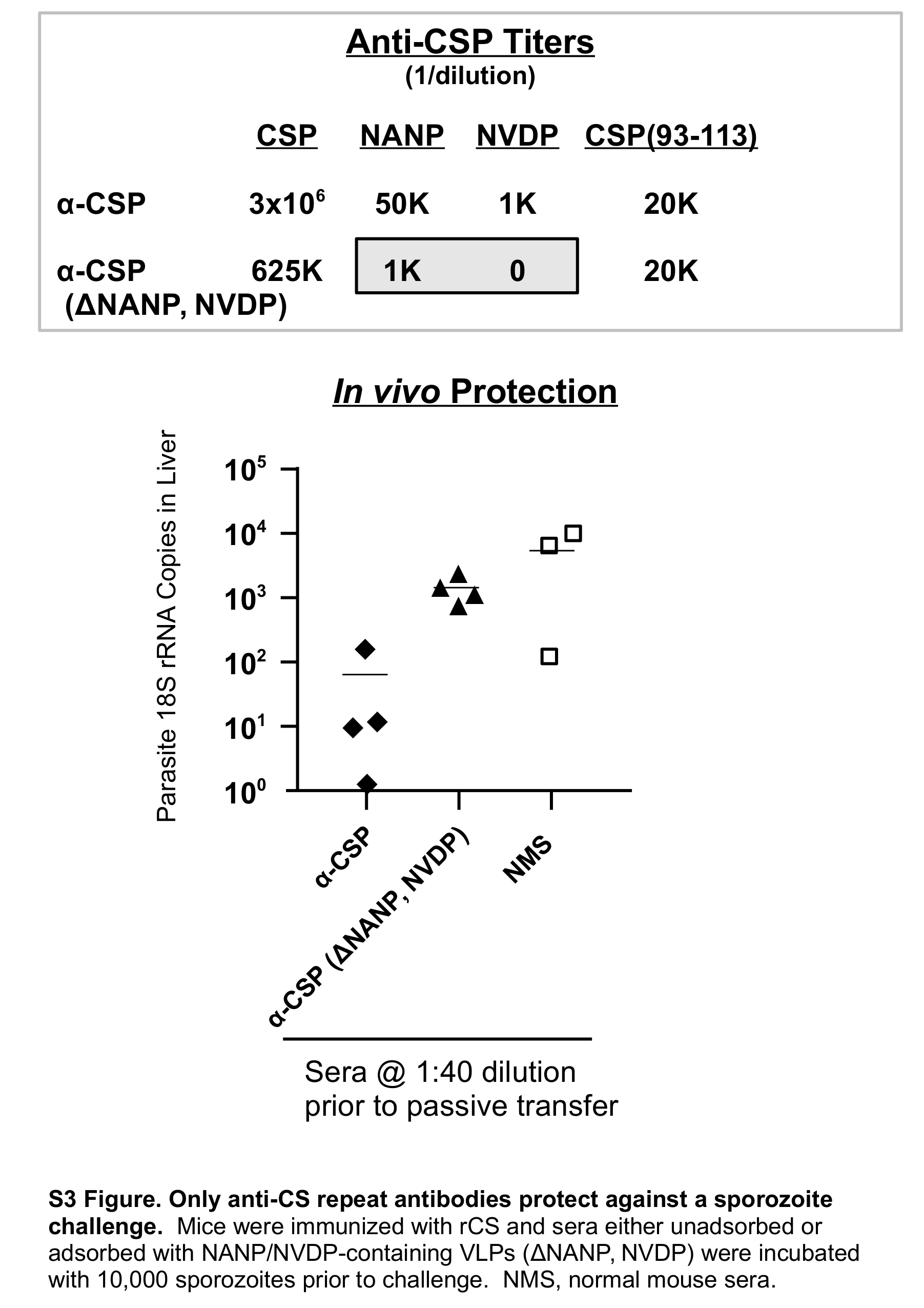

Supplement: S3 Fig — Mice were immunized with rCS and sera either unadsorbed or adsorbed with NANP/NVDP-containing VLPs (ΔNANP, NVDP) were incubated with 10,000 sporozoites prior to challenge. NMS, normal mouse sera. (TIF) [file pone.0124856.s003.tif]

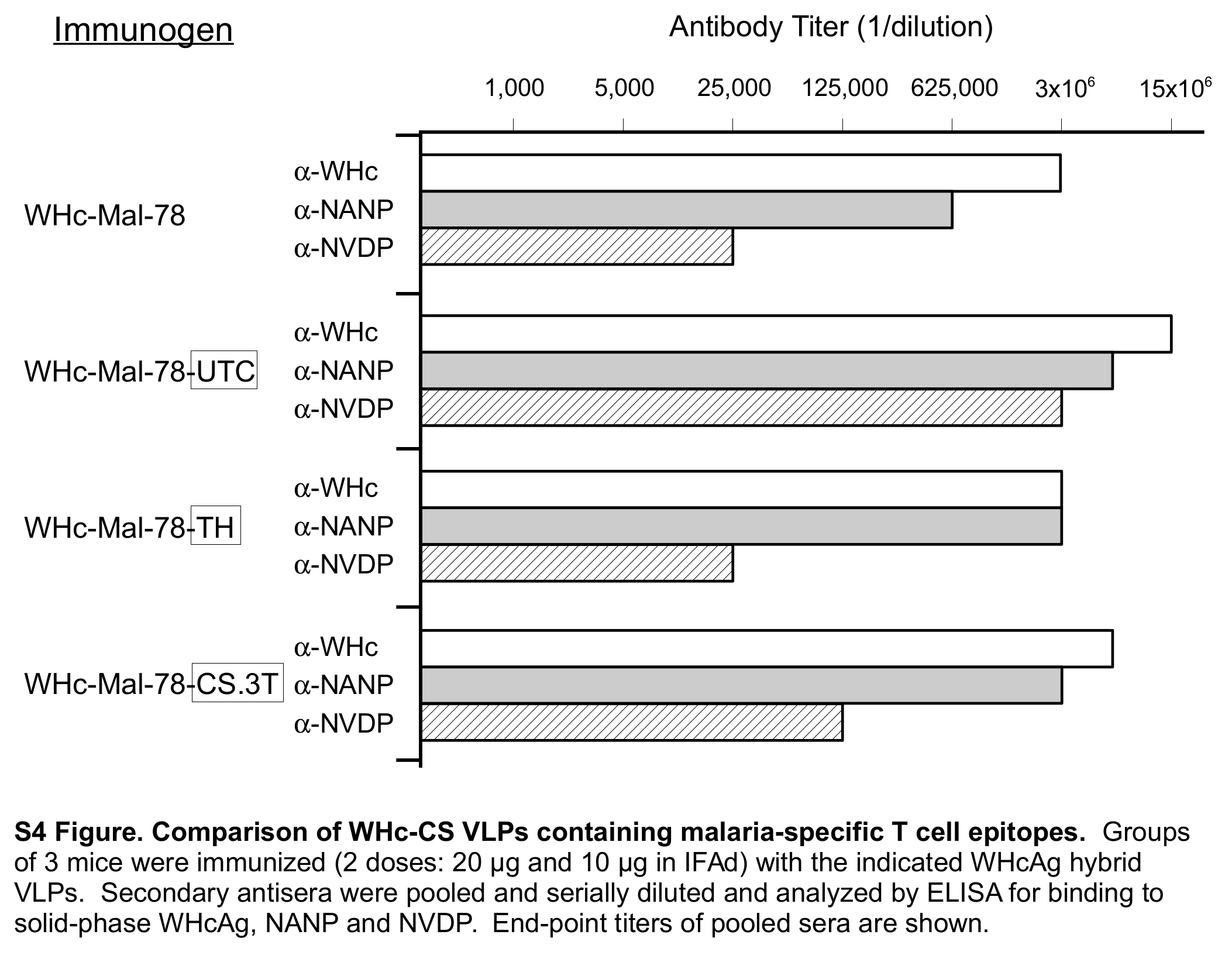

Supplement: S4 Fig — Groups of 3 mice were immunized (2 doses: 20 μg and 10 μg in IFAd) with the indicated WHcAg hybrid VLPs. Secondary antisera were pooled and serially diluted and analyzed by ELISA for binding to solid-phase WHcAg, NANP and NVDP. End-point titers of pooled sera are shown. (TIF) [file pone.0124856.s004.tif]

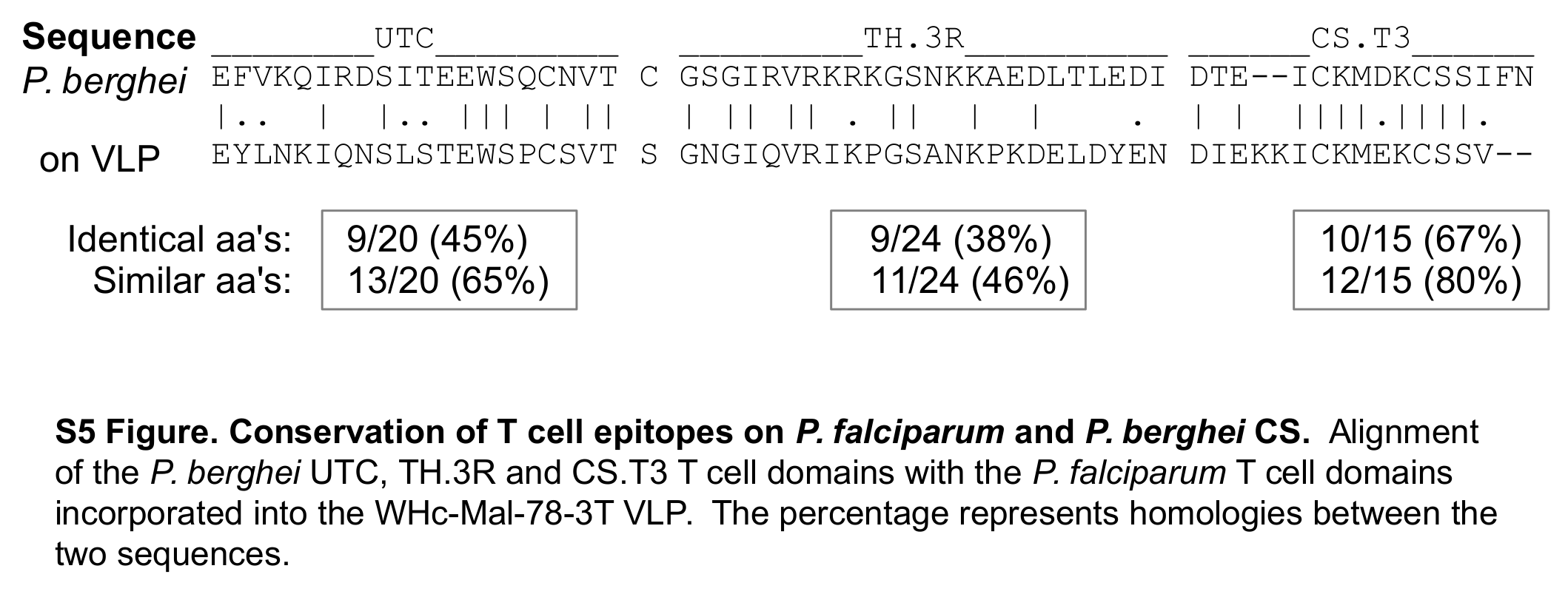

Supplement: S5 Fig — Alignment of the P. berghei UTC, TH.3R and CS.T3 T cell domains with the P. falciparum T cell domains incorporated into the WHc-Mal-78-3T VLP. The percentage represents homologies between the two sequences. (TIF) [file pone.0124856.s005.tif]
