# Supplementary material for: P. falciparum and P. vivax Epitope-Focused VLPs Elicit Sterile Immunity to Blood Stage Infections
Source: PLoS One. 2015 May 1;10(5):e0124856. doi: 10.1371/journal.pone.0124856 (PMC4416889; doi:10.1371/journal.pone.0124856)
Supplement: S1 Table — Mean endpoint dilution titers from 9–10 mice in each group are shown. (PDF) [file pone.0124856.s006.pdf]

Supporting Information Table S1. Kinetics of IgG Ab titers through primary immunization (1°) with WHc(C61S)-Mal-78-3T, at the boost (2°) and at 3 months post-challenge.

			Endpoint Titer (1/dilution)			
**Immunogen**	**Formulation**		**α-rCSP**	**α-NANP**	**α-NVDP**	**α-WHc**
**WHc(C61S)-**	Alum	1°	82.5K	43K	5.5K	125K
**Mal-78-3T**	Alum	2°	$4.7x10^6$	$4x10^6$	900K	$3.5x10^6$
		3 mo post-challenge	$2.2x10^6$	$1.6x10^6$	540K	875K
**WHc(C61S)-**	Alum+QS-21	1°	103K	68K	13.5K	125K
**Mal-78-3T**	Alum+QS-21	2°	$4.5x10^6$	$4.8x10^6$	$1.5x10^6$	$3x10^6$
		3 mo post-challenge	$1.25x10^6$	925K	242K	$1.7x10^6$
**WHc(C61S)-**	ISA-720	1°	183K	113K	17.8K	475K
**Mal-78-3T**	Alum	2°	$4.3x10^6$	$4.3x10^6$	$1.3x10^6$	$2.6x10^6$
		3 mo post-challenge	$1.25x10^6$	$1.1x10^6$	160K	708K
**WHcAg**	ISA-720	1°	0	0	0	625K
	Alum	2°	0	0	0	$11.7x10^6$
		3 mo post-challenge	na	na	na	na

Mean endpoint dilution titers from 9-10 mice in each group are shown.
