# Supplementary material for: P. falciparum and P. vivax Epitope-Focused VLPs Elicit Sterile Immunity to Blood Stage Infections
Source: PLoS One. 2015 May 1;10(5):e0124856. doi: 10.1371/journal.pone.0124856 (PMC4416889; doi:10.1371/journal.pone.0124856)
Supplement: S2 Table — Mean endpoint dilution Ab titers shown. 1°, primary; 2°, secondary antisera. IFA assay used Pb/Pv dry sporozoites. (PDF) [file pone.0124856.s007.pdf]

Supporting Information Table S2. Immunogenicity of WHc-Pv-78 VLPs.

				Antibody Titer (1/dilution)		
**Immunogen**	**Adjuvant**	**Dose**	**1°/2°**	**α-WHc**	**α-Pv1a**	**IFA**
**WHc-Pv-78**	Alum	100 µg	1°	80.5K	24.5K	-
n=9		100 µg	2°	$9.5x10^{6}$	$2.2x10^{6}$	8.1K
**WHc-Pv-78**	IFAd	20 µg	1°	250K	250K	-
n=3		10 µg	2°	$3x10^{6}$	$3x10^{6}$	-
**WHc-Pv-78**	IFAd	100 µg	1°	$3x10^{6}$	$9x10^{6}$	-
n=8		100 µg	2°	$6.5x10^{7}$	$1.5x10^{8}$	72.9K
**WHcAg**	IFAd	100 µg	1°	$6x10^{6}$	0	-
n=10		100 µg	2°	$2.3x10^{8}$	0	0
**Mab 2F2** (mg/ml)	-	-	-	0	$45x10^{6}$	-

Mean endpoint dilution Ab titers shown. 1°, primary; 2°, secondary antisera. IFA assay used Pb/Pv dry sporozoites.
